# Cel5I, a SLH-Containing Glycoside Hydrolase: Characterization and Investigation on Its Role in *Ruminiclostridium cellulolyticum*

**DOI:** 10.1371/journal.pone.0160812

**Published:** 2016-08-08

**Authors:** Nathalie Franche, Chantal Tardif, Julie Ravachol, Seddik Harchouni, Pierre-Henri Ferdinand, Romain Borne, Henri-Pierre Fierobe, Stéphanie Perret

**Affiliations:** Aix Marseille Univ, CNRS, LCB, Marseille, France; Institut National de la Recherche Agronomique, FRANCE

## Abstract

*Ruminiclostridium cellulolyticum* (*Clostridium cellulolyticum*) is a mesophilic cellulolytic anaerobic bacterium that produces a multi-enzymatic system composed of cellulosomes and non-cellulosomal enzymes to degrade plant cell wall polysaccharides. We characterized one of the non-cellulosomal enzymes, Cel5I, composed of a Family-5 Glycoside Hydrolase catalytic module (GH5), a tandem of Family-17 and -28 Carbohydrate Binding Modules (CBM), and three S-layer homologous (SLH) modules, where the latter are expected to anchor the protein on the cell surface. Cel5I is the only putative endoglucanase targeting the cell surface as well as the only putative protein in *R*. *cellulolyticum* containing CBM17 and/or CBM28 modules. We characterized different recombinant structural variants from Cel5I. We showed that Cel5I has an affinity for insoluble cellulosic substrates through its CBMs, that it is the most active endoglucanase on crystalline cellulose of *R*. *cellulolyticum* characterized to date and mostly localized in the cell envelope of *R*. *cellulolyticum*. Its role *in vivo* was analyzed using a *R*. *cellulolyticum cel5I* mutant strain. Absence of Cel5I in the cell envelope did not lead to a significant variation of the phenotype compared to the wild type strain. Neither in terms of cell binding to cellulose, nor for its growth on crystalline cellulose, thus indicating that the protein has a rather subtle role in tested conditions. Cel5I might be more important in a natural environment, at low concentration of degradable glucose polymers, where its role might be to generate higher concentration of short cellodextrins close to the cell surface, facilitating their uptake or for signalization purpose.

## Introduction

Plant cell walls contain numerous polysaccharides among which cellulose is the most abundant. It is constituted of linear chains of glucosyl residues linked though β-1,4 glycosidic bonds, and packed in a crystalline arrangement highly resistant to hydrolysis [1, 2]. The conversion of cellulose into biofuel is a promising alternative to fossil energy-based fuels, however the biological breakdown into fermentable sugars remains one of the most challenging steps in the development of an industrial process. Among the organisms able to grow on such substrates, *Ruminiclostridium cellulolyticum* (formerly known as *Clostridium cellulolyticum* [3]) is a mesophilic gram positive anaerobic bacterium producing multi-enzymatic complexes called cellulosomes which are assembled in the extracellular medium [4]. These complexes degrade plant cell wall polysaccharides into soluble sugars which are subsequently imported and metabolized by the bacterium. The genome of *R*. *cellulolyticum* encodes sixty-two cellulosomal enzymes typically carrying a dockerin module [5]. This module allows them to be incorporated into the cellulosomal complexes through high affinity interaction with the cohesin modules of the cellulosomal scaffolding protein (CipC) [6]. The catalytic modules of the plant cell wall degrading enzymes belong mostly to the glycoside hydrolase (GH) and to a lesser extent, to the pectate lyase, and the carbohydrate esterase families, as classified by the CAZY database (http://www.cazy.org/) [5, 7].

In addition to the genes encoding cellulosomal enzymes, twelve other additional genes are predicted to encode non-cellulosomal secreted glycoside hydrolases. Five of them contain SLH domains which are typically found in proteins forming the S-Layer of many bacteria [8]. These domains are composed of approximately 50 amino acids and were shown to bind to peptidoglycan or secondary cell wall polymers [9, 10, 11]. This might suggest that these five enzymes could be localized at the cell surface in *R*. *cellulolyticum*. Sequence analysis indicates that their catalytic modules belong to different glycoside hydrolase families: a GH18 putative chitinase (gene at locus Ccel_0643), two GH10 putative xylanases (genes at loci Ccel_2319 and Ccel_2320), a GH43 putative β-xylosidase/α-L-arabinofuranosidase (gene at locus Ccel_3240), and a GH5 previously called Cel5I (gene at locus Ccel_0428) [12, 13].

Cel5I is a multi-modular protein. In addition to its GH5 and SLH domains, Cel5I also comprises a tandem of CBM17 and CBM28. Both CBMs belong to the type B CBMs, as classified by Boraston and coworkers [14, 15, 16]. Both are structurally related and display a β-sheet topology with a long open groove accommodating a single glycan chain rather than a crystalline surface like mostly found in cellulose [14, 16, 17, 18]. They were reported to bind to distinct and non-overlapping sites on non-crystalline cellulose [19].

The GH5 family is characterized by a (α/β)_8_ fold, and is one of the largest GH families. GH5 are heterogeneous in terms of substrate specificities (e.g. cellulase, mannanases, xylanase, xyloglucanase). This large family has been divided in fifty-one subfamilies according to phylogenetic analysis [20]. Some subfamilies are monospecific, *ie* the enzymes exhibits a single activity whereas others are polyspecific. According to the CAZY database, the GH5 catalytic domain of Cel5I belongs to the subfamily 2, in which most of the characterized members exhibit an *endo*-β-1,4-glucanase activity [20]. The genome of *R*. *cellulolyticum* encodes another six GH5 enzymes. Unlike Cel5I, they all contain a dockerin domain allowing them to integrate in cellulosomes. Among them two endoglucanases, Cel5A and Cel5D, and a mannanase, Man5K, were previously characterized [21, 22, 23].

A former study of *cel5I* expression in *R*. *cellulolyticum* revealed that the gene is transcribed as a monocistronic transcription unit and regulated through a carbon catabolite repression mechanism [13]. The expression level of *cel5I* was reported to be ten times higher when *R*. *cellulolyticum* is grown on cellulose compared to cellobiose as substrate. But otherwise, a recent transcriptomic approach to examine expression levels of *R*. *cellulolyticum* genes, showed that *cel5I* expression was not observed under various carbon sources, suggesting the gene might be expressed at a basal level [24].

Cel5I is unique in *R*. *cellulolyticum* since it is the sole SLH-containing protein predicted to hydrolyze cellulosic substrates, and the only GH harboring a CBM17 and/or a CBM28. In the present work, we characterized enzymatic and binding properties of Cel5I, and investigated its role in *R*. *cellulolyticum*.

## Results

### Domain organization of Cel5I

**Fig 1:**
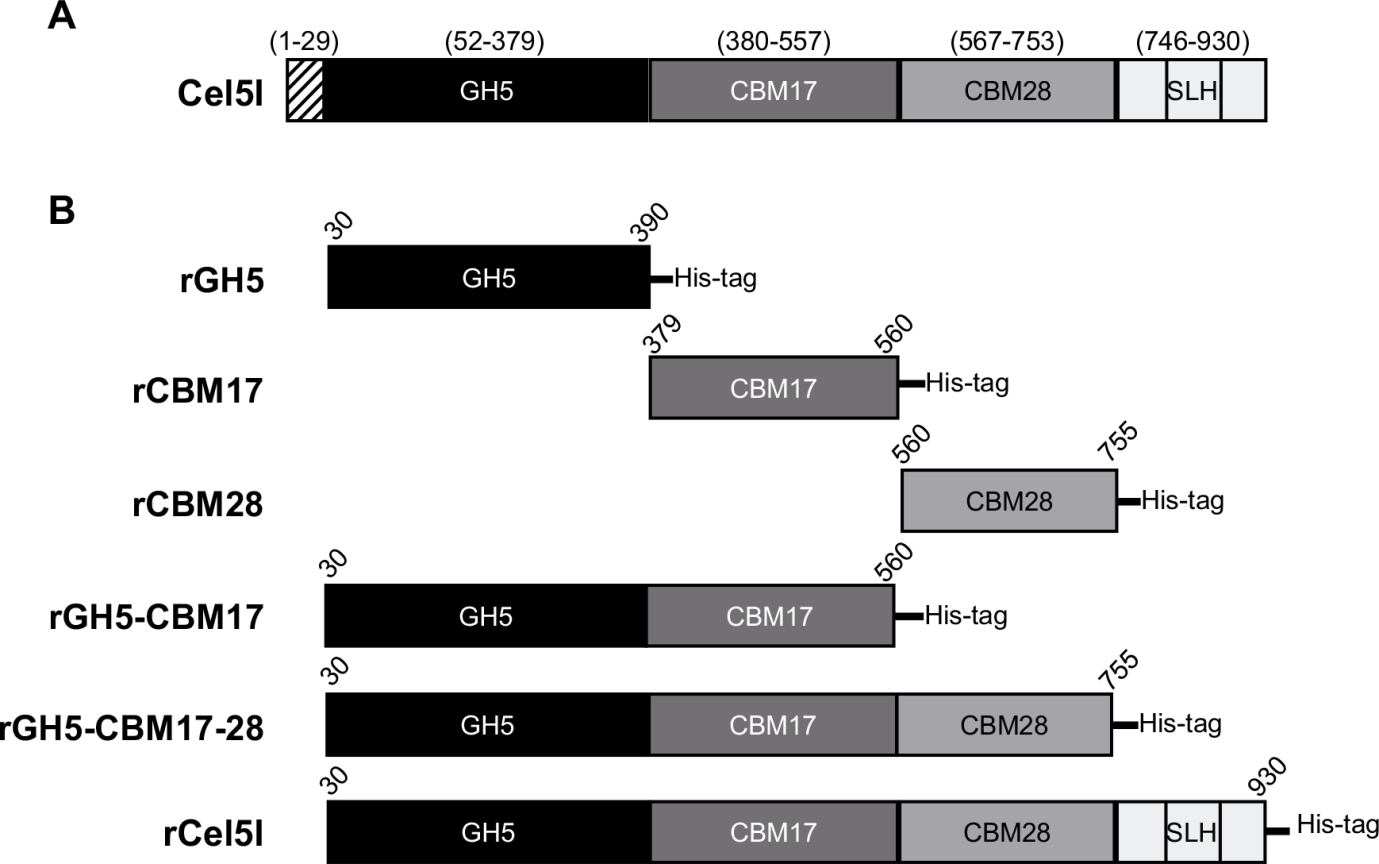
Domain organization of Cel5I and recombinant proteins produced. **A.** Prediction of domains and their boundaries in Cel5I from *Ruminiclostridium cellulolyticum*, according to Signal P, Superfamily and Prosite. Numbers above the protein correspond to the first and last amino acids of each predicted module according to the sequence of the full-length molecule. **B.** Modular organization of recombinant proteins produced in the present study. Numbers above the protein correspond to the first and last amino acids of each recombinant protein according to the sequence of the full-length protein.

The gene encoding Cel5I is located at locus Ccel_0428. Downstream and in the opposite direction, is located gene *cel44O* encoding a cellulosomal endocellulase of family GH44 [25], while the gene upstream is annotated to encode a resolvase. Full length Cel5I has a theoretical mass of 101.6 kDa and is composed of a signal sequence (29 first amino acids), followed by a GH5 catalytic module, a CBM17/CBM28 tandem, and three SLH domains (Fig 1). The CBMs share 17% identity, what is in the range of previously reported CBM17/CBM28 tandems found in other proteins [17]. Interestingly proteins highly similar to Cel5I are encoded in the genome of related clostridia, sharing a high level of the sequence identity all along the protein sequence: *Ruminiclostridium papyrosolvens strain C7* (WP_020815883.1) (93% sequence identity), *Ruminiclostridium* sp BNL1100 (WP_014312969.1) (93% sequence identity), *Ruminiclostridium josui* (WP_024834501.1) (89% sequence identity), *Clostridium termitidis* (WP_040761884.1) (72% sequence identity) and *Clostridium cellobioparum* (WP_034860559.1) (72% sequence identity).

**Fig 2:**
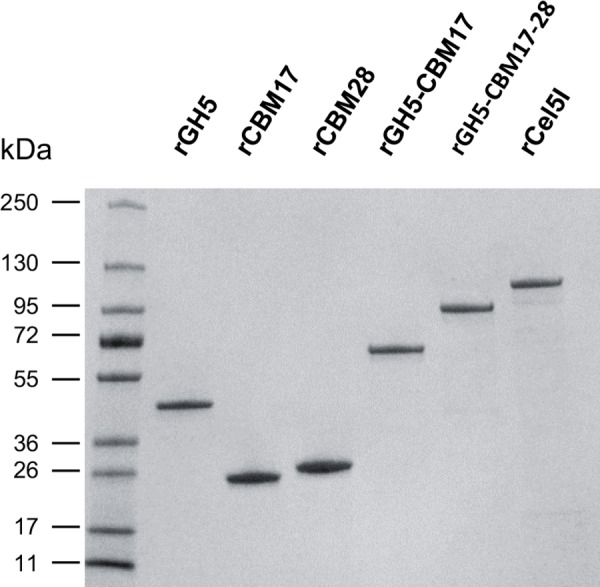
Purified recombinant derivatives of Cel5I. Purified proteins (2 μg) were loaded on SDS-PAGE, and Coomassie blue stained. Migration of recombinant proteins was in good agreement with theoretical molecular mass rGH5 (41 kDa); rCBM17 (20.6 kDa); rCBM28 (22,5 kDa); rGH5-CBM17 (59 kDa), rGH5-CBM17-28 (80 kDa), and rCel5I (99 kDa).

To examine the role(s) of the various domains of Cel5I, six different recombinant proteins tagged with a hexa-histidine sequence at their C-terminus were produced in *Escherichia coli* BL21(DE3): the full length Cel5I called rCel5I (99 kDa), the GH5 catalytic module only, called rGH5 (41 kDa), two truncated forms containing the GH5 catalytic module and the first CBM called rGH5-CBM17 (59 kDa), or containing both CBMs termed rGH5-CBM17-28 (80 kDa), CBM17 or the CBM28 alone, named rCBM17 (20.6 kDa) and rCBM28 (22.5 kDa) respectively. The various recombinant proteins were purified and their purity checked by SDS-PAGE (Fig 2). A single major band migrating at the expected mass was observed for each protein. Binding characteristics and the activity of these proteins on various substrates were tested.

### Protein binding to polysaccharides

**Fig 3:**
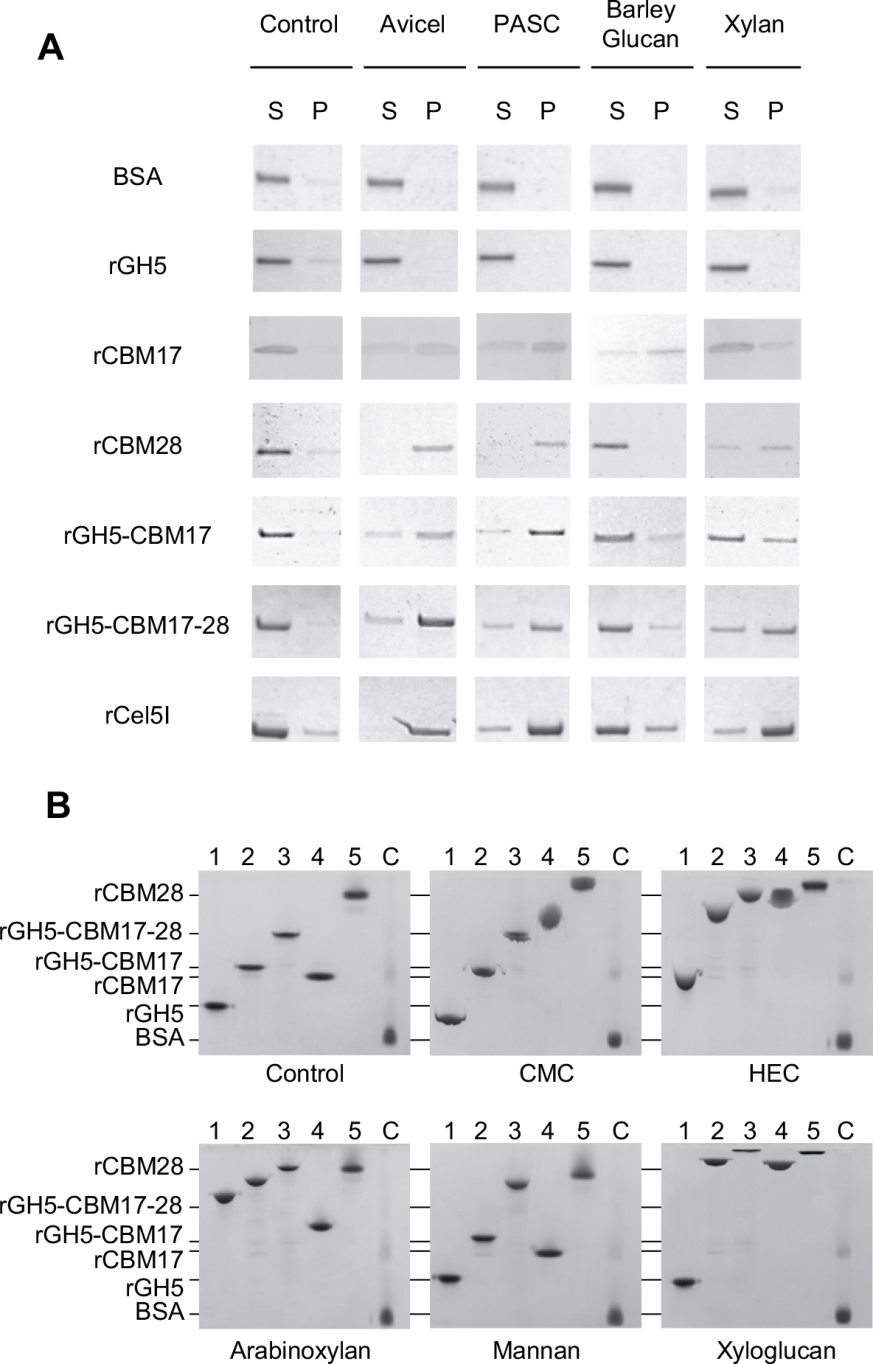
Interactions of recombinant derivatives of Cel5I with various substrates. **A.** Recombinant proteins were mixed with or without (control) insoluble substrates during one hour. After centrifugation, the bound proteins found in the pellet (P), and the unbound proteins found in the supernatant (S) were analyzed by SDS-PAGE. BSA was used as the negative control. **B.** Recombinant proteins were loaded on native gel electrophoresis containing no (control) or 0.1% soluble substrate CMC, HEC, arabinoxylan, mannan or xyloglucan. Delayed migration of the protein compared to control gel reflects an interaction with the polysaccharide. BSA was used as the negative control. The proteins loaded were: 1, rGH5; 2, rGH5-CBM17; 3, rGH5-CBM17-28; 4, rCBM17; 5, rCBM28; c, BSA.

Binding capacities were first determined on insoluble substrates such as amorphous cellulose (PASC), crystalline cellulose (Avicel), oat spelt xylan and barley glucan (Fig 3A). After incubation with the PASC and Avicel, the CBM-containing variants were generally detected in larger amounts in the polysaccharide-containing pellet, reflecting their ability to interact with insoluble cellulose. They were found to bind to a lesser extent to xylan, and barley glucan. CBM17 and CBM28 showed differences in their binding abilities. CBM28 seems to bind more specifically to cellulosic substrates (Avicel and PASC) compared to the other substrates, whereas the CBM17 can bind to any of the tested substrates but less strongly. The binding to soluble polysaccharides such as carboxy-methyl cellulose (CMC), 2-hydroxylethyl cellulose (HEC), xyloglucan, mannan and arabinoxylan was also analyzed using native gel shift assay (Fig 3B). The migration of the rCBM17 was altered with arabinoxylan as well as the GH5 and CBM17-containing Cel5I derivatives, but not rCBM28. No shift was observed for both rCBMs with mannan while the migration of rCBM17 and rCBM28 was altered with CMC, HEC and xyloglucan, indicating that both CBMs may bind to these substituted polysaccharides. The absence of the SLH domains did not change the binding capacities, similar results were obtained with variant rGH5-CBM17-28 and rCel5I. Thus the C-terminal SLH domains are not involved in the binding to cellulosic substrates.

### Activity of the various engineered forms of Cel5I

The enzymatic activity of Cel5I derivatives was assayed on various cellulosic and hemi-cellulosic substrates (Table 1). The proteins rGH5, rGH5-CBM17, rGH5-CBM17-28, were active on β-1,4 linked only glucosyl chains such as CMC, PASC and Avicel, as well as on Barley glucan, which contains β-1,4 / β-1,3 linked glucosyl residues. The enzymes also had modest but detectable activity on xyloglucan and xylan.

**Table 1:** Specific activities of the various forms of Cel5I on different substrates.

	**CMC**	**PASC**	Avicel*	**Barley Glucan**	Xyloglucan*	Xylan*	**pNPCb**
**rGH5**	527 ± 5	42 ± 6	50 ± 8	467 ± 17	16 ± 1	18 ± 5	82 ± 2
**rGH5-CBM17**	482 ± 45	139 ± 22	120 ± 6	416 ± 48	16 ± 3	14 ± 3	74 ± 0.5
**rGH5-CMB17-C28**	513 ± 67	139 ± 33	142 ± 7	344 ± 31	18 ± 4	26 ± 6	83 ± 3
**rCel5I**	512 ± 8	144 ± 17	143 ± 12		17 ± 5		88 ± 1

Specific activity values are given in micromoles min^-1^ of product released per μmol of enzyme, as the mean of at least triplicate, excepted for Avicel*, xyloglucan* and xylan where the values are given in μM of released products after 24 h of incubation with 100 nM enzyme. “-”means not tested.

Specific activity were calculated based on reducing sugars measurement at 37°C with 0,35% (w/v) of substrate, excepted for CMC (1%) and pNP-cellobioside (0,1%). Assays were performed with 5 nM enzyme on CMC and barley glucan, 10 nM on PASC, 100 nM on pNPC, xylan, xyloglucan and Avicel.

The catalytic module alone (rGH5) and the other forms of Cel5I, containing either one or two CBMs, showed comparable activity on soluble sugars like CMC, xyloglucan or pNP-cellobiose, indicating that Cel5I does not require CBMs to hydrolyse these substrates. The activity on insoluble substrates such as barley glucan or xylan, for which weak binding of the CBM-containing forms of Cel5I (rGH5-CBM17, rGH5-CBM17-28 and rCel5I) was observed, was in the same range in variants with or without CBMs. This suggests that CBMs do not significantly influence activity on these substrates. Activity on PASC and Avicel was shown to be improved in the presence of at least CBM17, since all CBM-bearing forms are about three times more active on Avicel and PASC than the catalytic module alone (rGH5). The presence of two CBMs versus one has no or moderate influence on the activity of the enzyme on these two substrates; indeed the addition of the second CBM increases activity up to 18%. Furthermore, no significant differences were observed between rCel5I and rGH5-CBM17-28, thus indicating that the SLH domains located at the C-terminus of rCel5I do not contribute to its catalytic activity.

**Fig 4:**
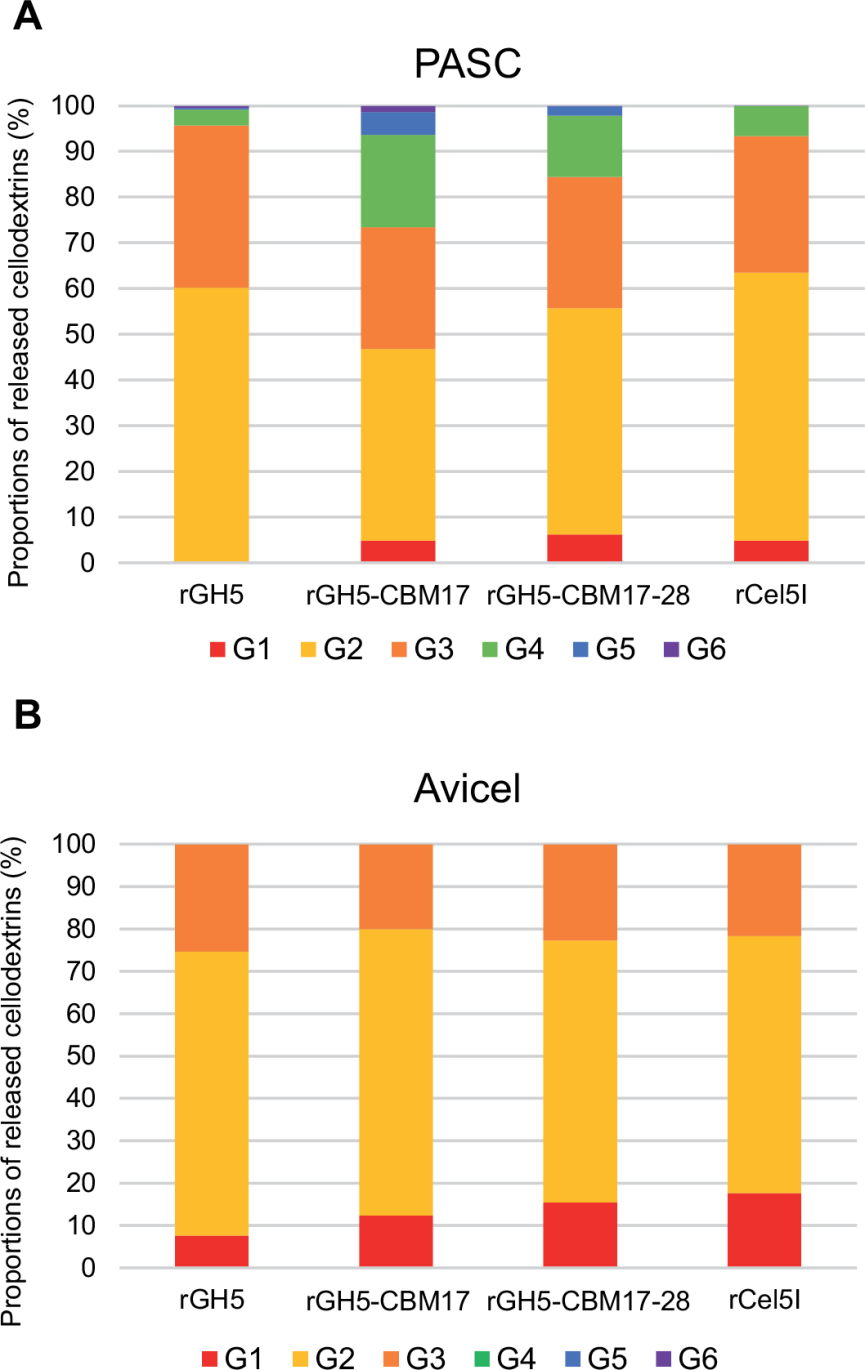
Analysis of the cellodextrins released by the various Cel5I forms on insoluble celluloses. Soluble sugars generated by 0.1 μM of enzyme after 15 min of incubation on amorphous cellulose PASC (A) or 24 h on crystalline cellulose Avicel (B), were identified and quantified by HPAEC-PAD.

After 15 minutes of incubation with PASC, the enzymes released mostly cellobiose (40 to 60% of the sugars detected), followed by cellotriose (25%) and cellotetraose (5 to 20%), suggesting an endo type mode of action of Cel5I (Fig 4A). This was confirmed through a viscosimetric assay, showing a clear rise in relative fluidity of CMC substrate treated with GH5 (S1 Fig).

After 24 hours of incubation with Avicel, larger amounts of shorter dextrins were detected compared to PASC. Cellobiose was the main sugar released (70% of the dextrins), whereas cellotriose and glucose represented 20% and 10%, respectively (Fig 4B). This may be due to the fewer accessible sites available on Avicel compared to PASC, leading to a more complete hydrolysis of initially released long dextrins.

### Examination of the putative role of Cel5I in *R*. *cellulolyticum*

To study the role of Cel5I in *R*. *cellulolyticum*, we constructed a *cel5I* mutant strain named MTL*cel5I*, using the Clostron mutagenesis tool developed by Heap and coworkers [26]. Analysis of chromosomic DNA of this mutant by PCR and Southern blot confirmed a single insertion of the intron in *cel5I* (S2 Fig).

**Fig 5:**
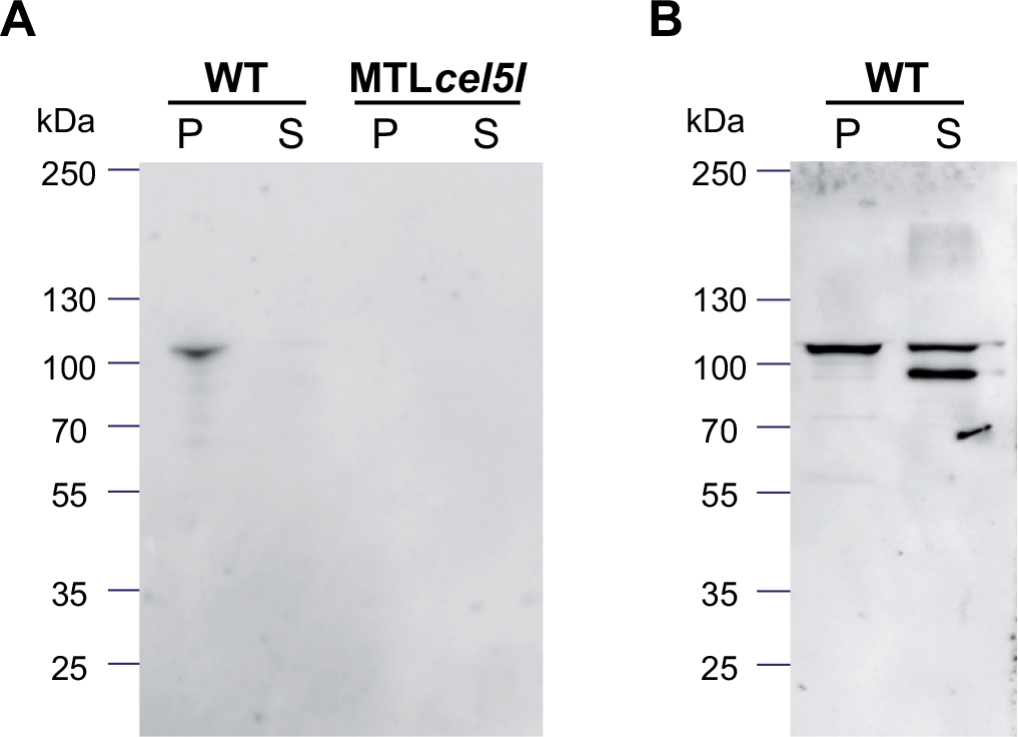
Detection of Cel5I in *R*. *cellulolyticum* strains. Wild-type and MTL*cel5I* strain were probed with anti-CBM28 antibody. Aliquot was taken from a culture on cellobiose substrate at the mid (A) or late (B) exponential phase of growth, and centrifuged to separate the cells and the supernatant. Cell fraction and 12% TCA precipitated supernatant fraction corresponding to the same culture volume were subjected to SDS-PAGE. After transfer onto nitrocellulose membranes, membranes were probed with the antiserum raised against CBM28.

**Fig 6:**
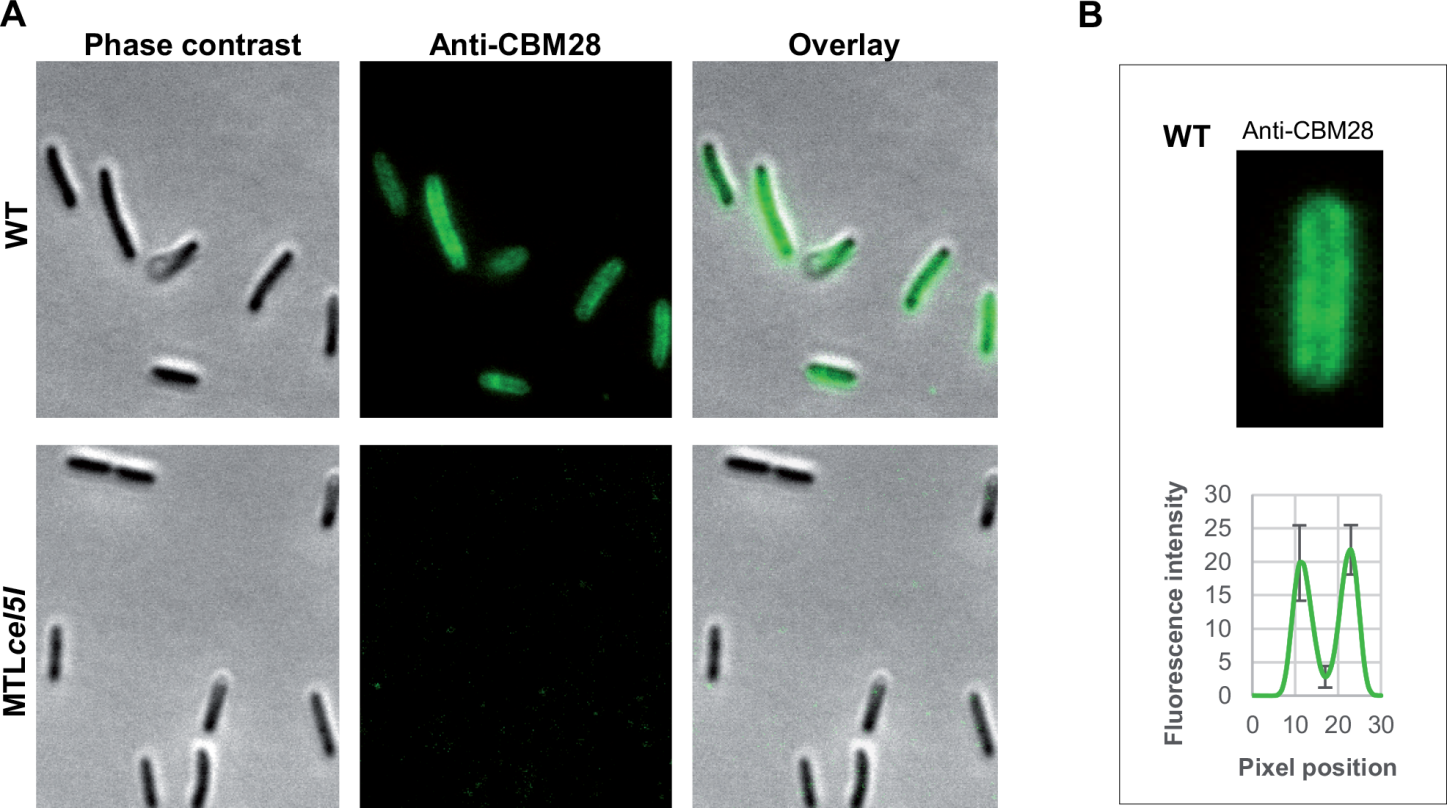
Immunolocalization of Cel5I in *R*. *cellulolyticum* strains. **A**. Cel5I localization was analyzed by fluorescent microcopy on wild-type and MTL*cel5I* cells. Cells were grown on cellobiose and probed with anti-CBM28 serum, and then anti-Rabbit IgG, Hilyte Fluor^Tm^ 488-labeled. Phase contrast and fluorescence images were taken and overlaid. **B.** Representative cell fluorescence image, and analysis of fluorescence intensity through the cell body. The graph represents the mean of ten cells.

Cel5I has a signal peptide at its N-terminus that allows the protein to be secreted and harbors three SLH domains probably localizing the enzyme at the cell surface. To investigate the localization of Cel5I we used an antiserum raised against rCBM28.We analyzed the cell pellet and the supernatant of a wild-type culture at mid- and late- exponential phase of growth on cellobiose (Fig 5). Cel5I was mostly detected in the cellular fraction at its expected size of approximatively 100 kDa, at the mid-exponential phase of growth (Fig 5A). At the end of the exponential phase (Fig 5B), two different forms of Cel5I were detected in the supernatant: a full length and a truncated form. This suggests that the protein is secreted, tethered to the cell and detached partly from the cell during the growth. *R*. *cellulolyticum cel5I* mutant grown on cellobiose was also probed. As expected no signal was detected with the mutant strain, confirming the absence of Cel5I (Fig 5A). We further analyzed the localization of Cel5I by fluorescence microscopy using the same antiserum, for wild type and *cel5I* mutant strains grown on cellobiose (Fig 6). As expected, fluorescence was only observed with the wild type strain. A signal of greater intensity was observed on the sides of the cell bodies, matching with the envelope (Fig 6B). Overall these results are consistent with a localization of Cel5I at the cell surface.

It has already been reported that cellulolytic bacteria bind to cellulose [27, 28, 29, 30]. We have shown in a previous study that in *R*. *cellulolyticum*, CipC is only partly responsible for cell adhesion to filter paper [30], suggesting that other mechanism(s) may participate to bring the bacteria close to the cellulosic substrate. As Cel5I is located at the surface of the cell and able to bind to cellulose, we tested the contribution of Cel5I to the binding of the *R*. *cellulolyticum* cells to filter paper. MTL*cel5I* mutant was found to bind to cellulose at the same level (72% ± 5) as wild type (71.5% ± 4), showing that the inactivation of *cel5I* has no measurable impact on cell adhesion to filter paper.

Growth of the mutant and the wild type strain on soluble and insoluble substrates was also measured. On soluble cellobiose, similar doubling times were observed for the wild type strain (6.5 h ± 1) and the mutant strain (6.3 h ± 0.6). On insoluble cellulose (Sigmacell), similar growth curves were also observed, either with rich or minimal medium, indicating that the mutation of *cel5I* has no obvious impact on the growth of the bacterium under our experimental conditions (S3 Fig).

## Discussion

A set of GH5 enzymes with identical or very similar modular organization as Cel5I was previously studied: Cel5A from *Bacillus sp1139* (*Bs*Cel5A) and from *Clostridium josui* (CjCelA) exhibit identical domain arrangements to Cel5I [19, 31]; the enzyme Csac_0678 from *Caldicellulosiruptor saccharolyticus*, contains no CBM17 [32], and EngF from *Clostridium cellulovorans* lacks the SLH domains and CBM28 [33]. All these enzymes were shown to hydrolyze β-1,4 glycosidic linkage found in amorphous cellulose, CMC or Avicel [32, 33, 19]. But like Cel5I, Csac_0678 also hydrolyzes xylan and xyloglucan [32]. Specific activities of these enzymes, when available, are reported to be quite different from one enzyme to another. For example, EngF displays a specific activity of 1.7 IU/μmol on PASC, whereas the specific activity of Csac_0678 measured at 70°C was 200 IU/μmol. The latter is in a similar range as that of Cel5I measured at 37°C. In *R*. *cellulolyticum* the most characterized GH5 is the endoglucanase Cel5A which is composed of a catalytic and a dockerin module [21]. Comparison of released reducing sugars on Avicel in similar experimental assays indicates that Cel5I is approximately eight times more active on this substrate than Cel5A [34]. Furthermore, compared under similar experimental conditions to other GH9 cellulases of *R*. *cellulolyticum*, Cel5I is twice more active on Avicel than Cel9E, the most active of these enzymes [35]. Thus Cel5I can be considered to be the most efficient *R*. *cellulolyticum* cellulase on crystalline cellulose characterized to date.

Like in Cel5I, the CBM17 and CBM28 from BsCel5A or CjCel5A and the CBM28 from Csac_0678 were shown to bind to amorphous cellulose and/or Avicel, probably to its non-crystalline regions [19, 31, 32]. In our study, removal of both CBMs decreased substantially activity of Cel5I on Avicel and PASC. A similar observation was formerly reported for BsCel5A, where the absence of the CBMs led to a twofold reduction of the enzymes activity on amorphous cellulose [19]. In addition to their capacity to bind to cellulosic substrates, CBMs of Cel5I were also shown to bind to substituted celluloses, as well as to other types of polysaccharides such as barley glucan, xylan, or arabinoxylan even if not at equal extents. A similar specificity was reported for CBM17 and CBM28 of CjCel5A which bind to xylan and cellulose [31]. These binding characteristics and the subtle binding differences between both CBMs broaden the range of substrates recognized by Cel5I and might modulate the binding strength of Cel5I on these substrates.

Cel5I is an endoglucanase localized at the surface of *R*. *cellulolyticum*, and is well conserved among other cellulolytic mesophilic clostridia. Ozdemir *et al*. proposed that the role of the protein Csac-0678 constituted of a GH5, a CBM28 and three SLH domains was to bring *Caldicellulosiruptor saccharolyticus* close to its insoluble susbstrate [32]. In *R*. *cellulolyticum* it appears that Cel5I has no obvious influence on the binding of the bacteria to filter paper cellulose under our conditions tested. However, the binding properties of the CBMs of Cel5I might help to recognize other types of hemicellulose like xyloglucan or xylan. In *C*. *cellulovorans*, the SLH-containing protein EngE, was proposed to be indirectly involved in cell binding to cellulose. This cellulosomal enzyme was proposed to attach the cellulosomes to the cell surface with its C-terminal type I dockerin and its N-terminal SLH domains, thus mediating the binding of the cell to cellulose [36]. No EngE homolog was found in *R*. *cellulolyticum*, but its genome encodes 29 putative proteins containing SLH domains [8]. Four of these proteins also include CBMs which may bind to amorphous cellulose or xylan: WP_015925747 (CBM4-GH10-CBM9-3SLH, 159 kDa), WP_015925748 (2CBM4-GH10-CBM9-2SLH, 112 kDa)), WP_015924512 (CBM9-3SLH, 242 kDa) and WP_015926587 (GH43-4CBM6-3SLH, 220 kDa). Most of the other proteins comprise domains or sequences of unknown function [8]. They are candidates for putative binding abilities of *R*. *cellulolyticum* to cellulose and constitute also a source of new domains and functions to be explored.

No specific mechanism dedicated to attach the cellulosomes to the cell surface of *R*. *cellulolyticum*, has been reported [30]. Consequently, Cel5I might be the only endoglucanase localized at the cell surface of the bacterium. Some cell surface GHs (unrelated to Cel5I) were reported to be essential for signalization and/or utilization of polysaccharides degradation products. For example, utilization of glycogen by *Streptococcus pneumoniae* was dependent on the presence of SpuA, a cell surface glycogen degradation enzyme that reduces long chains of glycogen into shorter dextrins, allowing their subsequent assimilation [37]. Alternatively, in *Paenibacillus* sp.W-61, the cell surface xylanase Xyn5 was reported to act as a sensor for induction of xylanase-encoding genes expression, by delivering short xylodextrins in close proximity to the cell surface [38]. A similar essential role of Cel5I in *R*. *cellulolyticum* could not be established by our experiments. However, we cannot exclude that on more complex substrates in terms of polysaccharides diversity and/or in a more stringent environment, the presence of this enzyme at the cell surface may be advantageous. It has been reported that the expression of the *cel5I* in *R*. *cellulolyticum* is submitted to carbon catabolic repression regulation, and therefore induced when only scarce amounts of carbon nutrients are available [13]. For example at a very early stage of growth on cellulose, when *cel5I* expression level might be induced, this enzyme may be of importance in terms of binding, uptake or signalization, due to its quite high activity on and binding capability to amorphous and crystalline cellulose. The partial degradation of cellulosic chains by Cel5I in close proximity of the cell surface, might locally increase the concentration of short cellodextrins, favoring their uptake and/or facilitating sensing mechanisms in the initial states of cellulosome synthesis.

## Materials and Methods

### Strains, media and vectors

Strains used in this study are reported in S1 Table. *Escherichia coli* strains were grown at 37°C in Luria-Bertani medium supplemented with appropriate antibiotics (100 μg.mL^-1^ of ampicillin or 35 μg.mL^-1^ chloramphenicol). *R*. *cellulolyticum* H10 ATCC 35319 [39] and mutants were grown anaerobically at 32°C on basal medium [40] supplemented with either 2 g.L^-1^ cellobiose or 5 g.L^-1^ Sigmacell20 (Sigma-Aldrich, Saint Louis, MO). For minimal medium yeast extract was removed from basal medium. When necessary, thiamphenicol (5 μg.mL^-1^) or erythromycin (2.5 μg.mL^-1^) was added to the medium. Colonies of recombinant *R*. *cellulolyticum* mutant strain were isolated under the anaerobic atmosphere of a glove box (N_2_-H_2_, 95:5 [vol/vol]), on solid basal medium supplemented with 2 g.L^-1^ of cellobiose, 15 g.L^-1^ of agar, and 2.5 μg.L^-1^ of erythromycin. Plates were incubated in anaerobic jars under 2 x10^5^ Pa of an N_2_-CO_2_ (80:20 [vol/vol]) atmosphere.

Growth on cellobiose-supplemented basal medium was followed by monitoring optical density at 450 nm over time. When cultured on 5 g.L^-1^ Sigmacell, growth was monitored by measurement of the total protein content as described previously [41].

Vectors used in this study are reported in S1 Table. The expression plasmid pET22b(+) (Novagen, Madison, WI) was used for the production in *E*. *coli* BL21 (DE3) of the recombinant derivatives from Cel5I. A derivative of pMTL007 was used for inactivation of *cel5I* gene in *R*. *cellulolyticum*.

### Construction of *cel5I* mutations in *R*. *cellulolyticum*

Gene inactivation in *R*. *cellulolyticum* was performed using the ClosTron technology as described by Heap *et al*., 2007 with minor modifications [26, 30]. The integration sites in the target genes and the primers used to retarget the Ll.LtrB intron in the pMTL007 (IBS, EBS1d and EBS2) allowed antisens intron integrations (S2 Table). They were generated by the Perutka algorithm implemented at http://ClosTron.com. Specific *cel5I* target primer IBS, EBS1d and EBS2 and the universal primer EBS universal were used to produce a fragment by overlapping PCR using pMTL007 as the matrix. The fragments and the recipient pMTL007 were subsequently digested by BsrGI and HindIII and ligated, creating the pMTL007*cel5I*.

The vectors were methylated *in vitro* with MspI methylase prior to be transferred in *R*. *cellulolyticum* by electro-transformation as previously described [42, 43], and thiamphenicol resistant clones carrying replicative pMTL007-*cel5I* were selected. In a second step, the integration event was selected in erythromycin-containing basal medium after induction with 1 mM isopropyl-β-D-thiogalactopyranoside (IPTG). The modified strain interrupted in the gene at locus Ccel_0428 was called MTL*cel5I*.

Southern blot was performed as described in Blouzard *et al*, 2010 [44]. EcoRV-digested genomic DNAs which were purified from the MTL*cel5I* mutant and wild type *Ruminiclostridium* strain, were hybridized with a labelled probe targeting erythromycin marker gene.

### Cloning of the genes encoding recombinant forms of Cel5I in *E*. *coli*

The primers used in this study are presented in S2 Table. All recombinant proteins were designed to be fused with a sequence of 6 histidine residues at their C-terminus (Fig 1). All genes were obtained by PCR performed on the genomic DNA of *R*. *cellulolyticum* using the forward and reverse primer pairs: ID6/IRcat for the production of rGH5, ID6/IR4 for the production of rCel5I, ID6/IR5 for the production of rGH5-CBM17-28, ID6/IR7 for the production of rGH5-CBM17, IDCBM17 and IR7 for the production of rCBM17, and ID7/IR5 for the production of rCBM28. The amplicons were subsequently digested with NdeI and XhoI and cloned in a NdeI-XhoI linearized pET22b(+) thereby generating the corresponding pET-derivatives. These vectors were used to transform the BL21 (DE3) strain to produce the corresponding recombinant proteins.

### Production and purification of recombinant proteins

Recombinant *E*. *coli* BL21 (DE3) was grown at 37°C with shaking to an optical density at 600 nm of 1.0. IPTG was then added at a final concentration of 200 μM, and the cultures of *E*. *coli* BL21 (DE3) were incubated overnight under shaking at 20°C. The cells were then harvested by centrifugation for 15 min at 6000 *g* and broken in a French press. After centrifugation of the crude extract (10 min, 4°C, 10000 *g*) the His-tagged proteins present in the supernatant were loaded on a column of Ni-nitrilotriacetic acid superflow resin (Qiagen, Hilden, Germany) equilibrated with 30 mM Tris-HCl (pH 8), and eluted using the same buffer supplemented with 60 mM imidazole. After concentration by ultrafiltration (Vivaspin 10 kDa cutoff, Sartorius, Germany), the proteins were further purified by an anion exchange chromatography (Hi-trap Q-sepharose, GE Healthcare, Uppsala, Sweden). rCel5I protein was purified using Avicel cellulose in place of Ni-nitrilotriacetic acid superflow resin and was eluted using ultrapure water. Fractions of interest were pooled, dialyzed, and concentrated in 30 mM Tris-HCl (pH 8) by ultrafiltration (Vivaspin 20, 10 kDa cutoff). The absorbance at 280 nm was measured and the protein concentration was determined using their specific extinction coefficient.

### Polyacrylamide gel electrophoresis and Western blot analysis

Sodium dodecyl sulfate-polyacrylamide gel electrophoresis (SDS-PAGE) was performed using a vertical electrophoresis system using precast gels 4–15% acrylamide (Biorad). Gels were stained with Coomassie blue or were electroblotted onto nitrocellulose membranes (Hybond-ECL, GE Healthcare,). Membranes were probed with polyclonal rabbit antibodies raised against CBM28 protein of Cel5I. The antibodies were obtained as previously described [45]. Primary antibodies were detected using anti-rabbit horseradish peroxidase conjugate (Promega, Madison, WI) and a chemiluminescent substrate (Millipore, Billerica, MA).

### Analysis of cell and supernatant proteins from *R*. *cellulolyticum* strains

*R*. *cellulolyticum* and MTL*cel5I* strains were grown in rich medium supplemented with cellobiose and then harvested by centrifugation at mid or late exponential phase of growth. Proteins of the supernatant (0.5 mL) were precipitated with ice cold-TCA 12% final concentration (v/v). After centrifugation, the pellet was washed twice with acetone, dried, and solubilized with denaturing loading buffer. Cell pellet was boiled before SDS-PAGE analysis.

### Enzymatic activities

Substrates were mixed with enzymes in 20mM Tris Maleate buffer pH 6.0, 1mM CaCl2, 0.01% azide. The concentrations of the substrates were as follows: 3.5 g/L for Avicel microcrystalline cellulose (PH101, Fluka, Buchs, Switzerland), phosphoric acid swollen cellulose (PASC), oat spelt xylan (Sigma-Aldrich), xyloglucan (Megazyme, Wiclow, Ireland), barley glucan (Megazyme) and 1% CarboxyMethyl Cellulose (CMC) (medium viscosity, Sigma). PASC was prepared as previously described [46], and the activity assays were performed as formerly reported [35]. Briefly, the substrates were mixed with a final enzyme concentration varying from 5 nM to 100 nM at 37°C. At specific intervals, aliquots were taken and cooled on ice. Reducing sugars concentration was measured directly (soluble substrates) or on the supernatant after a centrifugation of 10 min at 4°C and at 10,000 *g*, (insoluble substrates) using the Park and Johnson ferricyanide method with glucose or xylose as standard [47]. One unit of activity (International unit, IU) corresponds to 1 μmol of D-glucose equivalent released per min. Activities on crystalline cellulose Avicel, xyloglucan and xylan, are given in μM equivalent D-glucose released after 24 hours. Released sugars were in some cases also analyzed by high performance anion exchange chromatography coupled with pulsed amperometric detection (HPAEC-PAD).

HPAEC-PAD analyses of the released soluble sugars were performed using a Dionex ICS 3000 (Sunnyvale, CA) equipped with a pulsed amperometric detector as previously described [35]. Samples (200 μL) were mixed with 50 μL of 0.5 M NaOH, and 25 μL were applied to a Dionex CarboPacPA1 (4 x 250 mm) column, preceded by the corresponding guard column (4 x 50 mm). Sugars were eluted with the buffers 0.1 M NaOH (A) and 0.5 M sodium acetate with 0.1 M NaOH (B) using the following procedure: isochratic separation (5 min, 95% A + 5% B), separation gradient (8 min, 10–37% B), column wash (2 min, 99% B) and subsequent column equilibration (2.5 min, 95% A + 5% B).

Activity on *p-*nitrophenyl-β-D cellobioside (pNPCB), (Sigma) at 1 g/L in 50 mM potassium phosphate buffer, pH 7.0, 0.01% (w/v) azide was measured by incubating at 37°C 1 mL of substrate solution with 10 μL of enzyme at 10 μM and monitoring the pNP release at 400 nm. One IU corresponds to 1 μmol of *p-*nitrophenol (Sigma) released per min.

### Viscosimetric assays

Viscosimetric assays were performed as previously described [35]. The flow time of 3.5 g/L CMC (in 20 mM Tris-maleate pH 6.0, 1 mM CaCl_2_, 0.01% (w/v) sodium azide) mixed at 37°C with appropriate quantities of enzyme for different incubation times was monitored. 1-mL samples were boiled for 15 min, and the fluidity was measured at room temperature. The relative fluidity ΔF was determined as [To/(T-To)]-[To/(To’-To)], where To, To’ and T correspond to the flow time of buffer, the flow time of CMC solution without enzyme, and the flow time of substrate with enzyme, respectively. In parallel, the reducing sugars content of the samples was determined using the Park and Johnson method [47].

### Protein binding assays

The binding of the various proteins to insoluble polysaccharides was examined by incubating 100 μL at 1.5 μM of protein with 5 mg of Avicel microcrystalline cellulose, PASC, barley glucan or oat spelt xylan in 50 mM potassium phosphate buffer (pH 7.0) in final volume of 100 μL during one hour at 4°C under gentle shaking. After centrifugation (10 min, 10,000 *g*) the pellet was washed twice with the same buffer and samples of the pellet fraction (bound proteins) and the supernatant (unbound proteins) were analyzed by SDS-PAGE.

The binding to soluble substrates like xyloglucan, CMC, HEC (Sigma, St Louis, MO), Locust Bean Gum galactomannan (LBG) (Fluka, Buchs, Swiss) and wheat arabinoxylan (Megazyme, Wicklow Ireland) was monitored by loading 3 μg of proteins of interest in 8% acrylamide gel ProSieve (Lonza, Basel, Switzerland) containing 0 or 0.1% (w/v) of the soluble substrates.

### Immuno-labeling, epifluorescence microscopy, and GFP distribution into cells

A sample of 1 mL of culture at OD_450_ of 0.4 was pelleted (10 min at 6,000 *g*) and washed three times with 1 mL of phosphate buffered saline (PBS). 100 μL of cells were dropped on Poly-L-lysine coated microslide channel (Ibidi) and incubated for 1 h. The cells were fixed for 20 min at room temperature with 3.7% formaldehyde (Euromedex) in PBS. The cells were then washed three times with PBS before a short incubation with methanol, and followed by an incubation with 0.2 mg/mL lysozyme, 1 mM EDTA in 10 mM Tris HCl pH8. The cells were subsequently washed with PBS and saturated with 100 μL PBS- 5% BSA for labeling. After three washes with 100μL of PBS, the cells were incubated for 1 h with the primary anti-CBM28 antiserum (1/100). Cells were afterwards washed, and incubated with the secondary antibody (1/400, anti-Rabbit IgG, Hilyte Fluor^Tm^ 488-labeled, Eurogentec) for 1 h in the dark. Observation was carried out with an inverted epifluorescence microscope (Nikon TiE-PFS) coupled to a CCD camera (Hamamatsu OrcaR2). A 100x NA1.3 oil PhC objective and adequate filter set (Semrock HQ GFP) were used to record contrast phase and green fluorescence images. Analysis was performed using Image J software to combine contrast phase and GFP pictures for every field. GFP distribution of single cells cross section was measured with the plugin RGBProfilesTool. Data represent the mean of ten individual cells.

### Cell adherence assay

Binding assays protocol was based on the previously described protocol [30]. *R*. *cellulolyticum* cells were mixed at exponential growth phase to an optical density of 0.5 at 450 nm. Cell suspension (2 mL) was incubated with a strip of filter paper, saturated with 4% BSA. After 1 hour of incubation with gentle agitation, the OD_450_ of the supernatant was measured. Adhesion percentage was deduced from OD_450_ measurement of an assay, compared with the OD_450_ measurement of a control where no filter paper was added. The reported values presented are the mean of 3 triplicates performed in at least 2 independent experiments.

### Bioinformatics analysis

Predictions of domains from Cel5I amino acids sequence were performed using on line tools: Interpro (http://www.ebi.ac.uk/interpro/), Signal P (http://www.cbs.dtu.dk/services/SignalP/), Superfamily (http://supfam.cs.bris.ac.uk/SUPERFAMILY/index.html), CATH (http://www.cathdb.info/) and ScanProsite (http://prosite.expasy.org/scanprosite/) [48–52]. Amino acid sequences were compared with those in the NCBI database using the BLAST program [53] (http://blast.ncbi.nlm.nih.gov.gate1.inist.fr/Blast.cgi). Multiple sequence alignments were performed with the ClustalW2 program (http://www.ebi.ac.uk/Tools/msa/clustalw2/) [54].

## Supporting Information

S1 Fig Viscosimetric measurements. ΔF is the relative fluidity of the CMC with rGH5.
(PDF)

S2 Fig Analysis of the MTL*cel5I* strain. **A. Southern blot.** Genomic DNA purified from the wild type and MTL*cel5I* mutant strain was digested with EcoRV and tested with a labelled probe targeted to the erythromycin marker gene. A theoretical size of 7.8 kb was expected for the MTL*cel5I* genomic DNA lane. No signal was detected for wild type genomic DNA lane, in contrary to the native pMTL*cel5I* vector lane. This vector contains the erythromycin marker gene and was used as the positive control**. B. PCR screening of the integrant.** The region surrounding the insertion site in *cel5I* was amplified using the primers Cel5ID and Cel5IR. From the wild type strain the amplicon has an expected size of 240 bp and from MT*Lcel5I* strain the amplicon has an expected size of 1543 bp. The results indicate that the group II intron (1300 bp) is present in *cel5I*.
(PDF)

S3 Fig Growth curves on cellulose substrate. The wild type strain (square) and MTL*cel5I* mutant strains (triangle) were studied on rich medium (A) or on minimal medium (B) containing Sigmacell as growth substrate (5 g.L^-1^).
(PDF)

S1 Table Bacterial strains and vectors used in this study (PDF)

S2 Table Primers used in this study (PDF)
