# Supplementary figures and images for: Cel5I, a SLH-Containing Glycoside Hydrolase: Characterization and Investigation on Its Role in Ruminiclostridium cellulolyticum
Source: PLoS One. 2016 Aug 8;11(8):e0160812. doi: 10.1371/journal.pone.0160812 (PMC4976890; doi:10.1371/journal.pone.0160812)

**S1 Fig**

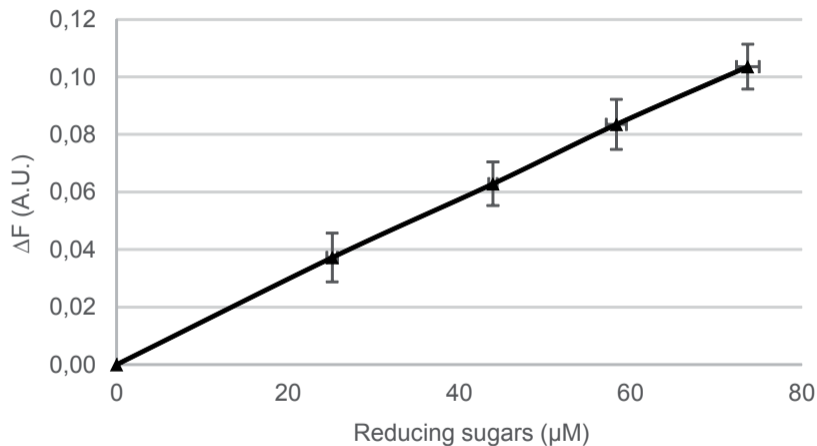

**Viscosimetric measurements.**

Supplement: S1 Fig — ΔF is the relative fluidity of the CMC with rGH5. (PDF) [file pone.0160812.s001.pdf]

## S2 Fig

**A**

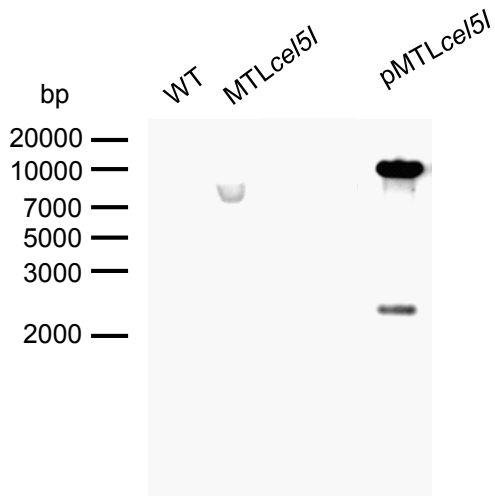

**B**

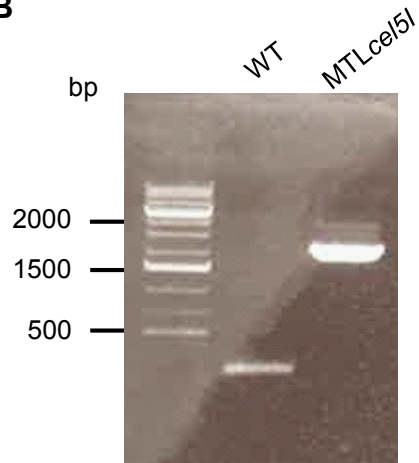

**Analysis of the MTLce/5l strain.**

**A. Southern blot. B. PCR screening of the integrant.**

Supplement: S2 Fig — A. Southern blot. Genomic DNA purified from the wild type and MTLcel5I mutant strain was digested with EcoRV and tested with a labelled probe targeted to the erythromycin marker gene. A theoretical size of 7.8 kb was expected for the MTLcel5I genomic DNA lane. No signal was detected for wild type genomic DNA lane, in contrary to the native pMTLcel5I vector lane. This vector contains the erythromycin marker gene and was used as the positive control. B. PCR screening of the integrant. The region surrounding the insertion site in cel5I was amplified using the primers Cel5ID and Cel5IR. From the wild type strain the amplicon has an expected size of 240 bp and from MTLcel5I strain the amplicon has an expected size of 1543 bp. The results indicate that the group II intron (1300 bp) is present in cel5I. (PDF) [file pone.0160812.s002.pdf]

# S3 Fig

**A**

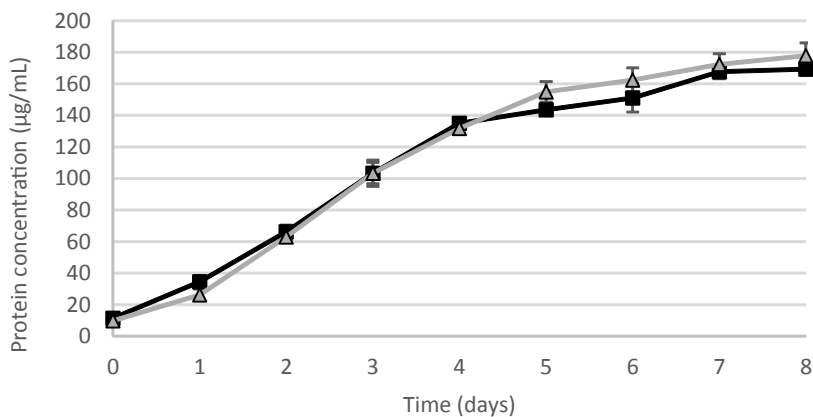

**B**

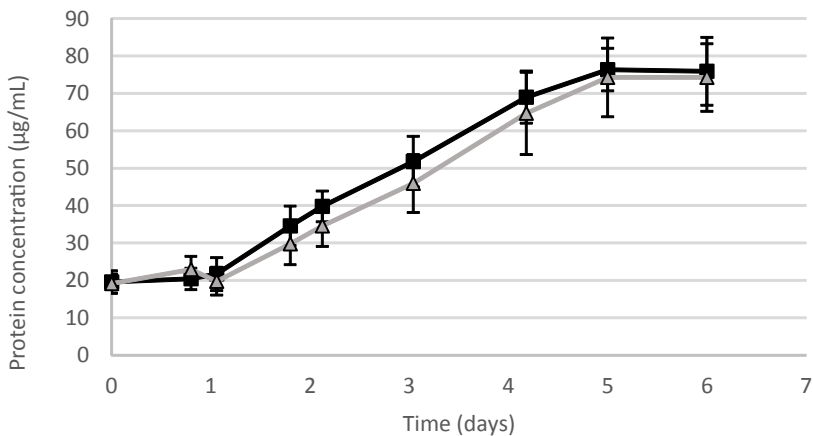

**Growth curves on cellulose substrate.**

Supplement: S3 Fig — The wild type strain (square) and MTLcel5I mutant strains (triangle) were studied on rich medium (A) or on minimal medium (B) containing Sigmacell as growth substrate (5 g.L-1). (PDF) [file pone.0160812.s003.pdf]
