# Supplementary material for: Cel5I, a SLH-Containing Glycoside Hydrolase: Characterization and Investigation on Its Role in Ruminiclostridium cellulolyticum
Source: PLoS One. 2016 Aug 8;11(8):e0160812. doi: 10.1371/journal.pone.0160812 (PMC4976890; doi:10.1371/journal.pone.0160812)
Supplement: S1 Table — (PDF) [file pone.0160812.s004.pdf]

S1 Table. Bacterial strains and vectors used in this study

Strain or plasmid	Relevant characteristics	Source or reference
***E. coli* DH5a**	F$^-$ *endA1 hsdR17*(rK$^-$ mK$^+$) *supE44 thi-1* λ^- *gyrA96 relA1Δ*(*lacZYA argF*) *U169* (Φ80 *lacZΔ*M15) *recA*	Roche Diagnostics
***E. coli* BL21(DE3)**	F$^-$ *ompT hsdS* (rB$^-$ mB$^-$) *gal dcm* (DE3)	Novagen
***R. cellulolyticum* H10**	Wild-type, ATCC35519 DSM 5812 (formerly *Clostridium cellulolyticum*)	DSMZ
R. cellulolyticum* MTL*cel5I	ATCC35319, *cel5I* ::intron, Erm^r	This study
**pET22b+**	*E. coli* expression vector; Ap^r	Novagen
**pETGH5**	pET22b+ derivative carrying the NdeI-XhoI fragment encoding the catalytic module of Cel5I	This study
**pETGH5-CBM17**	pET22b+ derivative carrying the NdeI-XhoI fragment encoding the catalytic module and the CBM17 of Cel5I	This study
**pETGH5-CBM17-28**	pET22b+ derivative carrying the NdeI-XhoI fragment encoding the catalytic module, the CBM17 and CBM28 of Cel5I	This study
**pETCel5IFL**	pET22b+ derivative carrying the bp NcoI-XhoI fragment encoding full length mature Cel5I	This study
**pETCBM28**	pET22b+ derivative carrying the NcoI-XhoI fragment encoding the CBM28 of Cel5I	This study
**pETCBM17**	pET22b+ derivative carrying the NcoI-XhoI fragment encoding the CBM28 of Cel5I	
**pMTL007**	*E. coli/Clostridium* shuttle vector (ColE1, pCB102)Ll.*ltr*Bintron (*erm*BtdRAM2) under the control of P*fac*, *ltr*A; Cm^r/Tm^r	[24]
**pMTL007*cel5I***	pMTL007 derivative targeting *cel5I* (locus Ccel_0428)	This study

Ap^r, ampicilline resistance ; Erm^r, erythromycin resistance ; Cm^r/Tm^r, chloramphenicol/thiamphenicol resistance.
