# Supplementary material for: Cel5I, a SLH-Containing Glycoside Hydrolase: Characterization and Investigation on Its Role in Ruminiclostridium cellulolyticum
Source: PLoS One. 2016 Aug 8;11(8):e0160812. doi: 10.1371/journal.pone.0160812 (PMC4976890; doi:10.1371/journal.pone.0160812)
Supplement: S2 Table — (PDF) [file pone.0160812.s005.pdf]

S2 Table. Primers used in the present study

Experiment	Name	Sequence	Features
**Targeted mutagenesis**	IBS*cel5I*	AAAAAAGCTTATAATTATCCTTAGCTGTCATAATGGTGCGCC CAGATAGGGTG	
	EBS1d*cel5I*	CAGATTGTACAAATGTGGTGATAACAGATAAGTCATAATGTC TAACTTACCTTTCTTTGT	
	EBS2*cel5I*	TGAACGCAAGTTTCTAATTTCGATTACAGCTCGATAGAGGAA AGTGTCT	
	EBS universal	CGAAATTAGAAACTTGCGTTCAGTAAAC	
**Mutant analysis**	Cel5ID	GGCAAACGTTCGTCTTTCAG	
	Cel5IR	CGGTGAATCAGCAGGTGTTA	
**Production in *E. coli***	ID6	CCCTATA<u>CAT**ATG**</u>GCGGAACCGGATAGCTC	NdeI is underlined, ATG boldface type
	ID7	CCCTATA<u>CAT**ATG**</u>ACTACCGTAGAAGCACCTGTTG	NdeI is underlined, ATG boldface type
	IDCBM17	GGGGG<u>CAT**ATG**</u>GATCGTGCTGAAAAAGAAGAGTTTAC	NdeI is underlined, ATG boldface type
	IR7	GTG<u>CTCGAG</u>ACGGTTTCCTGATACAGTTATATTG	XhoI is underlined
	IR5	GTG<u>CTCGAG</u>GCTGTCATTTTCGAACCTGAC	XhoI is underlined
	IR4	GTG<u>CTCGAG</u>TTTATGTAAACTGGTGTATATTTTATAAA	XhoI is underlined
	IRcat	TTTTTT<u>CTCGAG</u>ATCTGTCTTGGCTTCGAAAGCAT	XhoI is underlined
